# Correction: Dual targeting multiwalled carbon nanotubes for improved neratinib delivery in breast cancer

**DOI:** 10.1039/d5ra90025e

**Published:** 2025-03-19

**Authors:** Amr Selim Abu Lila, Rohini Bhattacharya, Afrasim Moin, Turki Al Hagbani, Marwa Helmy Abdallah, Syed Mohd Danish Rizvi, El-Sayed Khafagy, Talib Hussain, Hosahalli Veerabhadrappa Gangadharappa

**Affiliations:** a Department of Pharmaceutics, College of Pharmacy, University of Ha'il Ha'il 81442 Saudi Arabia; b Department of Pharmaceutics and Industrial Pharmacy, Faculty of Pharmacy, Zagazig University Zagazig 44519 Egypt; c Department of Pharmaceutics, JSS College of Pharmacy, JSS Academy of Higher Education and Research Mysuru 570015 India hvgangadharappa@jssuni.edu.in; d Department of Pharmaceutics, College of Pharmacy, Prince Sattam Bin Abdulaziz University Al-Kharj 11942 Saudi Arabia; e Department of Pharmaceutics and Industrial Pharmacy, Faculty of Pharmacy, Suez Canal University Ismailia 41522 Egypt; f Department of Pharmacology and Toxicology, College of Pharmacy, University of Ha'il Ha'il 81442 Saudi Arabia

## Abstract

Correction for ‘Dual targeting multiwalled carbon nanotubes for improved neratinib delivery in breast cancer’ by Amr Selim Abu Lila *et al.*, *RSC Adv.*, 2023, **13**, 24309–24318, https://doi.org/10.1039/D3RA04732F.

The authors regret an error in the TEM data in Fig. 3A. During peer review, reviewers requested that the authors include TEM data in their manuscript. As there is no TEM facility in their institution, the authors outsourced the TEM analysis. The authors have provided evidence that they outsourced the TEM analysis and were provided with the image used in Fig. 3A of the original manuscript by a third party. The corrected Fig. 3A is shown below.

**Fig. 3:**
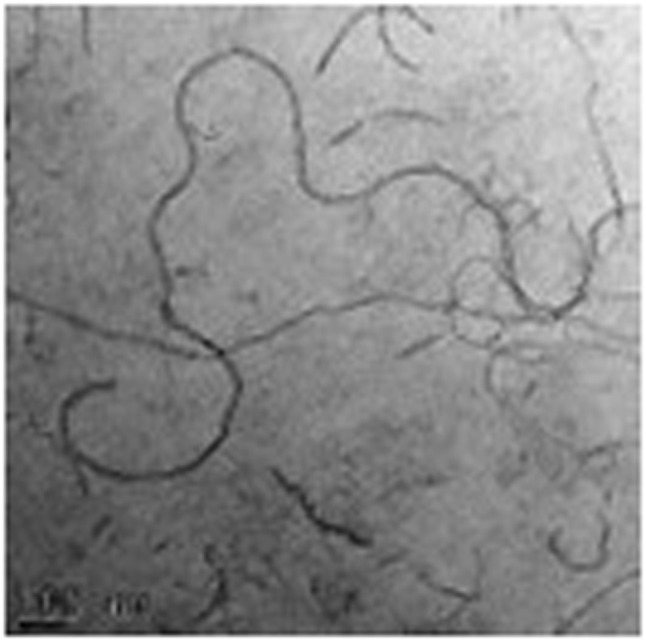
(A) TEM image of naked MWCNTs.

The authors regret an error in Section 2.3.5 ‘^1^H-Nuclear magnetic resonance’. In the first sentence, the instrument ‘Agilent 400 MHz FT-NMR spectrophotometer’ should have been written as ‘Agilent 400 MHz FT-NMR spectrometer’. In the second sentence, the solvent used was *d*-DMSO and not CDCl_3_. The corrected Section 2.3.5 is shown below.

**2.3.5. ^1^H-Nuclear magnetic resonance**

The instrument used for recording the ^1^H NMR spectrum was an Agilent 400 MHz FT-NMR spectrometer (Agilent Scientific Instruments, Santa Clara, CA, USA) operating at 400 MHz for protons. Samples (6 mg mL^−1^) were first dissolved in *d*-DMSO by keeping it at 50 °C overnight followed by vortex mixing for several minutes. With the deuterated solvent, *i.e.*, *d*-DMSO, the sample was scanned in the NMR tubes using tetramethylsilane as an internal standard.

An independent expert has viewed the corrected Fig. 3a and the changes to Section 2.3.5 and confirmed that they are consistent with the discussions and conclusions presented.

The Royal Society of Chemistry apologises for these errors and any consequent inconvenience to authors and readers.

